# Toll-Like Receptor 2 Modulates Pulmonary Inflammation and TNF-α Release Mediated by *Mycoplasma pneumoniae*

**DOI:** 10.3389/fcimb.2022.824027

**Published:** 2022-03-17

**Authors:** Ming Chen, Huan Deng, Yue Zhao, Xueqing Miao, Haiyan Gu, Ying Bi, Yifan Zhu, Yun Guo, Shuang Shi, Jiejing Xu, Deyu Zhao, Feng Liu

**Affiliations:** ^1^ Department of Respiratory Medicine, Children’s Hospital of Nanjing Medical University, Nanjing, China; ^2^ Department of Respiratory Medicine, Shanghai Children's Hospital, Shanghai Jiao Tong University, Shanghai, China; ^3^ Department of Respiratory Medicine, The Affiliated Wuxi Children’s Hospital of Nanjing Medical University, Wuxi, China

**Keywords:** *Mycoplasma pneumoniae*, neutrophils, A549 cells, TNF-α, TLR2, MyD88, NF-κB

## Abstract

**Objectives:**

To investigate the roles that Toll-like receptors (TLRs) play in lung inflammation mediated by *Mycoplasma pneumoniae* (MP).

**Methods:**

The changes in TLRs and tumor necrosis factor alpha (TNF-α) in peripheral blood of children with *M. pneumoniae* pneumonia (MPP) were monitored, and the interactions of signaling molecules regulating TNF-α release in A549 cells and neutrophils after *M. pneumoniae* stimulation were investigated. In TLR2 knockout (TLR2-/-) mice, the levels of TNF-α in bronchial alveolar lavage fluid (BALF) and peripheral blood after mycoplasma infection and the pathological changes in the lung tissue of mice were detected.

**Results:**

TNF-α levels in peripheral blood of children with MPP were higher than those in non-infected children, and children with refractory MPP had the highest levels of TNF-α and TLR2. TNF-α secretion and TLR2, myeloid differentiation primary response 88 (MyD88) and phospho-p65(p-p65) levels were increased in stimulated cells. TNF-α secretion was suppressed upon siRNA-mediated TLR2 silencing. Pharmacological inhibition of nuclear factor-kappa B (NF-κB) and MyD88 effectively reduced TNF-α expression. Compared with wild-type mice, the TNF-α in serum and BALF decreased, and lung pro-inflammatory response was partially suppressed in TLR2-/- mice.

**Conclusion:**

We concluded that TLR2 regulates *M. pneumoniae*-mediated lung inflammation and TNF-α release through the TLR2-MyD88-NF-κB signaling pathway.

## Introduction

*Mycoplasma pneumoniae* is one of the most common pathogens causing community-acquired pneumonia in children, accounting for 10%–40% of cases (Ngeow et al., 2005; Liu et al., 2014; Jain et al., 2015). In recent years, the incidence of MPP in China has increased. The infection rate in children older than 5 years reportedly is 70% (Tsolia et al., 2004). In general, *M. pneumoniae* infection is self-limiting; however, in refractory *M. pneumoniae* pneumonia (RMPP) cases, clinical symptoms or imaging manifestations do not improve or even continue to progress. The prognosis of these children is unfavorable, and RMPP can lead to necrotizing pneumonia, occlusive bronchitis, and other diseases, causing a heavy economic burden on the family and society (Tamura et al., 2008).

The exact pathogenesis of RMPP remains unclear, and macrolide resistance, mixed infection, and immune disorders may be underlying drivers (Saraya et al., 2014; Deng et al., 2018; Bi et al., 2021), especially, the excessive immune response is considered to be one of the important contributors to multisystem involvement and refractory diseases caused by *M. pneumoniae* infection.

Several classes of pattern-associated molecular patterns (PRRs), including TLRs, Nucleotide-binding oligomerization domain-like receptors (NLRs) and Retinoicacid-inducible gene-like receptors (RLRs) recognize distinct microbial components and directly activate immune cells (Wicherska-Pawłowska et al., 2021). TLRs are transmembrane receptors, while NLRs and RLRs are intracellular molecules. *M. pneumoniae*-derived community-acquired respiratory distress syndrome (CARDS) can trigger NLRP3 (leucine-rich repeat protein 3) inflammasome activation and interleukin-1β (IL-1β) secretion in macrophages to regulate the inflammatory response after *M. pneumoniae* infection (Segovia et al., 2017). At the same time, lipoproteins were identified as an important inflammation-inducing factor in *M. pneumoniae* (Tian et al., 2014). TLRs are well-described PPRs that, upon activation, induce the expression of proinflammatory cytokines and recruit immune cells to the site of infection (Mukherjee et al., 2016). Many TLRs signal through the adaptor protein MyD88 to exert their effects on cytokine expression (Cannova et al., 2015). NF-κB is located at a pivotal position downstream of the TLR signal, and is involved in many physiological and pathological processes, such as the regulation of inflammatory molecules, apoptosis, stress responses, and tumor growth inhibition (Mitchell et al., 2016). The MyD88-dependent pathway is an important way for NF-κB activation by TLRs (Naiki et al., 2005). Activated NF-κB regulates the expression of inflammatory factors, such as IL-4, IL-6, IL-8 and TNF-α (Hardy et al., 2001; Gu et al., 2020), and triggers an inflammatory response. Studies have shown that the TLR/NF-kB pathway is closely related to the occurrence of lung diseases such as acute lung injury, chronic obstructive pneumonia, asthma, and lung cancer (Arora et al., 2019).

*M. pneumoniae* is a cell wall free microbe and its lipoproteins, which are anchored in the membrane, are exposed to immune cells, hence, it has been hypothesized that TLR2 is the most important TLR which participates in recognition of *M. pneumoniae (* Naghib et al., 2018). However, recent reports suggest that *M. pneumoniae* also induces inflammatory responses also in a TLR2-independent manner. Lima-Neto et al. (2014) reported a positive association between expression levels of TLR4 and acute myocardial infarction in the *M. pneumoniae* infected patients. Shimizu et al. (2014)demonstrated that *M. pneumoniae* related cytoadherence is associated with inflammatory responses up-regulation of TLR4. Some studies have shown that purified or synthesized lipoproteins of *Mycoplasma* species induce inflammation through TLR2 (Okusawa et al., 2004; Shimizu et al., 2005). And studies have confirmed that compared with the control population, TLR2 is more often highly expressed in the immune population of patients with autoimmune disease (Marks et al., 2021). The lack of TLR2 expression caused by mouse germline knockout usually leads to a lack or attenuation of autoimmune inflammation (Frasnelli et al., 2005; Devaraj et al., 2011). In infectious diseases, the same study found that after knocking out TLR2, the systemic inflammation caused by *Klebsiella pneumoniae* infection was reduced (Jeon et al., 2017). This suggests that TLR is an important receptor for *M. pneumoniae*, and TLR2 may be closely related to the excessive inflammation caused by *M. pneumoniae* infection.

Airway epithelial cells provide the first line of defense against *M. pneumoniae* by secreting a series of cytokines and inflammatory factors, including TNF-α, which induce the recruitment of inflammatory cells, leading to local inflammation, airway remodeling, airflow obstruction, emphysema, and a decline in lung function (Lai et al., 2019). Wang et al. (2014) showed that serum TNF-α levels in children with RMPP were significantly higher than those in children with non-RMPP. Neutrophils are important inflammatory cells, and polymorphonuclear neutrophils (PMNs) are significantly increased in BALF and peripheral blood of MPP patients (Fujishima and Aikawa, 1995; Soehnlein et al., 2017). Beck-Schimmer et al. (2005) reported that in a toxin-induced lung injury model, alveolar macrophages regulate neutrophil recruitment through TNF-α secretion. Our previous work has confirmed that neutrophils in the peripheral blood of children with MPP are significantly increased, especially in children with RMPP (Bi et al., 2021), so we guessed that neutrophils may play an important role in excessive inflammation, pneumonia, and lung injury.

There is a lack of TLR2 impact on the disease MPP. Our focus is on the role of TLR2 in MPP at the human and animal levels. We sought to systematically discuss the role of TLR2 in lung tissue damage and systemic inflammation from the perspective of disease phenotypes after *M. pneumoniae* infection.

## Methods

### Study Subjects

Thirty-six patients admitted to the Children’s Hospital Affiliated to Nanjing Medical University (from December 2017 to January 2018), including 9 RMPP patients, 15 non-RMPP patients, and 12 non-infected surgical patients (Children undergoing polypectomy), were enrolled in this study. All MPP patients tested positive for *M. pneumoniae* in nasopharyngeal aspirate (≥1.0 × 10^^4^ DNA copies). All patients in the trial tested had nasopharyngeal secretions negative for respiratory syncytial virus, influenza virus, adenovirus, parainfluenza virus by immunofluorescence technique and *Chlamydia trachomatis* by nucleic acid testing. Seven respiratory virus detection reagents (D3 Ultra TM Respiratory Virus Screening & ID Kit, Diagnostic Hybrids, USA) were used for detection. The subjects also had negative bacterial cultures of nasopharyngeal secretions and double-negative blood cultures. RMPP was diagnosed based on the following criteria: 1) prolonged fever for 7 days or more, 2) increasing cough and infiltrates in chest radiograph despite administration of appropriate antibiotics (Tamura et al., 2008). The 12 non-infected surgical patients were included as non-infected group.

Clinical characteristics of children with MPP were provided in **Table 1**. Peripheral blood samples (2 mL) were collected after admission and centrifuged at 1,000 × *g* for 10 min. Serum aliquots were stored at –80°C. The study was approved by the Ethics Committee of Nanjing Medical University, and written informed consent was provided by the parents of the children before sampling.

**Table 1:** Clinical characteristics of children with MPP.

	MPP (n = 15)	RMPP (n = 9)	*P*
Age (years)	4.60 ± 2.76	5.35 ± 2.11	0.492
Sex (male/female)	11/4	6/3	1.00
Duration of fever before hospitalization, days	4.93 ± 5.40	8 ± 3.64	0.15
Length of hospitalization, days	7.13 ± 2.42	8.89 ± 4.17	0.20
Hydrothorax, n (%)	0 (0%)	5(55.55%)	0.003
Atelectasis, n (%)	0 (0%)	5(55.55%)	0.003
WBC×10^^9^/L	9.45 ± 6.73	7.57 ± 3.23	0.442
Neutrophils, %	55.84 ± 18.22	53.12 ± 15.41	0.71
Platelets ×10^^9^/L	236.53 ± 73.09	334.56 ± 220.63	0.12
LDH, U/L	412.40 ± 164.29	533.88 ± 193.13	0.13
CRP, mg/L	16.85 ± 11.85	21.78 ± 34.50	0.64

MPP, Mycoplasma pneumoniae pneumonia; RMPP, refractory M. pneumoniae pneumonia; WBC, white blood cells; LDH, L-lactate dehydrogenase; CRP, C-reactive protein; Data are presented as mean ± standard deviation or number (percentage). P < 0.05 is statistically significant.

### Bacterial Strains and Culture Conditions

Standard *M. pneumoniae* strains originally purchased from the American Type Culture Collection (ATCC; 29342, M129-B7) were maintained at the Children’s Hospital of Zhejiang University Affiliated Hospital. The strain was cultured in a *mycoplasma* broth consisting of *mycoplasma* broth base, mycoplasma selective supplement G, 0.5% glucose, and 0.002% phenol red (Shi et al., 2019). The medium was refreshed every two days (1mL *mycoplasm* suspension was pipetted into the culture flask and 4ml fresh *mycoplasma* medium was added). After culture for 5 to 6 days, the color of the medium turns from red to yellow, indicating the *M. pneumoniae* is in a logarithmic growth phase.

### Cell Culture

A459 human alveolar epithelial carcinoma cells (ATCC) were cultured in DuIbecco’s modified eagIe’s medium (DMEM) (Nanjing Vicente Biotechnology Co., Ltd., Nanjing, China) supplemented with 10% fetal bovine serum (FBS) (Gibco, Grand Island, NYC, United States). Neutrophils were extracted from the peripheral blood samples within two hours after collection using a Human Neutrophil Isolation Kit (Tianjin Haoyang Biological Products Co., Ltd., Tianjin, China). The cells were cultured in Roswell Park Memorial Institute (RPMI)-1640 medium (Nanjing Vicente Biotechnology Co., Ltd., Nanjing, China) containing 10% FBS in a 5% CO2 incubator at 37°C. Neutrophil purity was determined by Wright-Giemsa staining.

### Cell Treatments

The neutrophil cells from the peripheral blood of each non-infected child were counted under the microscope and divided into two parts. One was left untreated as a non-infected group; The other one was treated with mycoplasma stimulation as a mycoplasma stimulation group. Approximately 10^^6^ cells per serving were seeded in a 6-well plate. *M. pneumoniae* was quantified by *M. pneumoniae* nucleic acid detection kit (Da An Gene Co., Ltd. Of Sun yat-sen University, Guangzhou, China). After the quantification of *M. pneumoniae* it was centrifuged with phosphate belanced solution (PBS) and re-suspended for washing twice, and finally re-suspended with RMPI-1640 medium to the concentration of 10^^8^/mL. In the *M. pneumoniae* stimulation group, 1mL MP suspension was added to the neutrophils at an infection rate of 100:1. In the non-infected group, 1mL RMPI-1640 medium was added to neutrophils. Then the cells were incubated for 6 hours at 37°C and 5%CO2 in a cell culture chamber.

The A549 cells were inoculated in 6-well plates with DMEM medium containing 10%FBS. When they grew to 50% of the floor area, 1mL 10^^8^/mL *M. pneumoniae* mixed suspension was added as the *mycoplasma* stimulation group. In the non-infected group, 1mL cell culture medium was added. In the NF-κB inhibition group, the culture medium was supplemented with 100 μmol/L Ammonium pyrrolidinedithiocarbamate (PDTC) (an inhibitor of NF-κB) (Sigma-Aldrich, St. Louis, Missouri, USA) for 1 hour before simultaneously adding the 1mL 10^^8^/mL *M. pneumoniae* mixed suspension. In the MyD88 inhibition group, A549 cells were inoculated into the 6-well plate and pretreated with cell culture medium containing 100 μmol/L NBP2-29328 (a MyD88 inhibitor peptide set) (Novus Biologicals, Colorado, USA) concentration for 24 hours. When the cells grew to 50% density, 1mL 10^^8^/mL *M. pneumoniae* mixed suspension was added for 12 hours.

### Laboratory Animals

The wild-type mice (BALB/c mice) (age, 6–8 weeks; weight, 18–20 g) were purchased from the animal core facility of Nanjing Medical University and TLR2-/- mice (age, 6–8 weeks; weight, 18–20 g) were gifted by Professor Chenyang Zhao from the School of Pharmacy, Ocean University of China. The mouse tail was used for gene identification to determine the knockout of TLR2: a single band was found at 334bp in TLR2-/- mice and at 499bp in wild-type mice (supply data). All laboratory animals in our study were fed and kept in a specific-pathogen-free (SPF) environment. Mice in the *M. pneumoniae* infected group were anesthetized and injected with 25μL (10^^7^CFU/mL) *M. pneumoniae* suspension into the trachea by slow nasal instillation, whereas mice in the normal control group were instilled with isometric sterile saline. Peripheral blood, BALF, and lung tissue were collected on the 4th day of infection. All experiments were carried out in accordance with the National Institute of Health Guide for the Care and Use of Laboratory Animals, and were approved by the Institutional Animal Care and Use Committee, Nanjing Medical University.

### Mouse Samples Collection

The mice were euthanized by injection of chloral hydrate, and the eyeballs were quickly removed to collect blood in a sterile EP tube. After blood sampling, 0.5mL of PBS containing 2.5% FBS was injected through the main trachea, and then gently sucked back. This was repeated three times to collect BALF in a sterile EP tube (recovery rate of 90%). Samples of the right lower lobe were collected in 4% formaldehyde solution, fixed, paraffin embedding, sectioned, and hematoxylin-eosin staining for histomathological analysis.

### Measurement of TNF-α Concentration

The TNF-α concentration was measured using commercially available TNF-α enzyme linked immunosorbent assay (ELISA) kits (RayBio, Atlanta, Georgia, United States). Absorbance was measured at 450 nm, and TNF-α concentration in the serum and BALF was calculated according to standard curves.

### Quantitative Real Time Polymerase Chain Reaction

RT-qPCR was conducted as described previously (Liu et al., 2008). Total peripheral blood neutrophil RNA was isolated with TRIzol^®^ reagent (Invitrogen, Carlsbad, California, United States) and reverse-transcribed into cDNA using random primers and HiScript^®^ Reverse Transcriptase (Nanjing Vicente Biotechnology Co., Ltd., Nanjing, China). qPCRs were run on an Applied Biosystems 7500 system (Thermo Fisher Scientific, Waltham, Massachusetts, United States) using gene-specific primers and AceQ^®^ qPCR SYBR^®^Green Master Mix (Low ROX Premixed) (Nanjing Vicente Biotechnology Co., Ltd., Nanjing, China). The primer sequences used for human TLR genes were as follows: *TLR1* 5′-GCCAGAATTCTGTAAGC-3′ (sense) and 5′-CAAGTACCTTGATCCTG-3′ (antisense), *TLR2* 5′-CTGTCTTTGTGCTTTCTG -3′ (sense) and 5′-GAAGAATGAGAATGGCAGC-3′ (antisense), *TLR4* 5′-CGGAGGCCATTATGCTATGT-3′ (sense) and 5′-TTCTCCCTTCCTCCTTTTCC-3′ (antisense), *TLR6* 5′-AGTGTGACTACCCAGAAAG-3′ (sense) and 5′-CAGATCCAAGTAGATGCAG-3′ (antisense). Target gene expression levels were detected and normalized to glyceraldehyde-3-phosphate dehydrogenase (GAPDH) mRNA levels and relative expression was determined using the 2^–ΔCt^ method (Youn et al., 2021).

### Western Blotting

To examine protein expressions of p-p65 and Myd88 in A549 cells, total protein extraction kit (Biyun tian biotechnology co., LTD., Shanghai, China) was used to extract A549 cell proteins (Feng et al., 2016). The protein content was determined by Bradford assay. Equal amounts of proteins (30 μg protein/lane) were electrophoresed using 12% sodium dodecyl sulfate polyacrylamide gels in a Tris/HCl buffer system, followed by electrophoretic transfer to a polyvinylidene difluoride microporous membrane (BioRad, Hercules, PA, USA). Subsequently, the membranes were sealed with 5% skim milk for 2 hours at room temperature and incubated with the appropriate primary antibodies overnight at 4°C: rabbit anti-mouse GAPDH, rabbit anti-mouse p-p65 and rabbit anti-mouse MyD88 (all purchased at Cell Signaling Technology, Boston, Massachusetts, USA). All antibodies were diluted 1/1000. Following 5 times washes of 6 min with Tris-buffered saline with Tween-20 (TBST), immunodetection was accomplished using appropriate horseradish peroxidase-linked secondary antibodies (Cell Signaling Technology, Boston, Massachusetts, USA) and enhanced chemiluminescence system. The Image Lab imaging system (Bio-Rad, California, USA) was used to conduct optical density scanning of the bands, determine the integral optical density value of each band, and conduct optical density scanning analysis.

### Flow-Cytometric Analysis

A549 cells were grown to near confluence in 6-well plates and treated with *M. pneumoniae* (MOI 1:100) for 12 h, then harvested after trypsin incubation. The cell suspension was centrifuged at 300 × *g* for 10 min, and the pelleted cells were resuspended in PBS (up to 10^^7^ nucleated cells in 80 µL PBS). Twenty microliters of FcR Blocking Reagent (Miltenyi Biotec Technology & Trading (Shanghai) Co., Ltd., Shanghai, China) was added to the cell suspension for 10 min. Then, 2.5 μL of APC-labeled anti-human TLR4 and 2.5 μL of PE-labeled anti-human TLR2 antibodies (eBioscience, California, USA) were added to the cell suspension, and the mixture was incubated in the dark at 2–8°C for 10 min. The cells were washed in 1–2 mL PBS and centrifuged at 300 × *g* for 10 min.

BALF was washed in 5mL PBS, then centrifuged at 300 × *g* for 10 min. The centrifuged cells were resuspended in 100μL PBS and incubated with 10µL of FITC-Ly6G and APC-CD45 (Biolegend, California, USA) for 30min at room temperature in the dark. Finally, the cells were washed in 5 mL PBS and centrifuged at 300 × *g* for 10 min.

Obtained A549 cells and cells in BLAF were resuspended in 300μL PBS and then detected by flow cytometry.

### Small Interfering RNA-Mediated Gene Knockdown

Specific human TLR2 small interfering RNA (siRNA) and control siRNA were purchased from RIBO Biotechnology (Guangzhou ruibo technology co. LTD, Guangzhou, China). A549 cells at 30%–50% confluence were transfected with TLR2 or control siRNA using ribo*FECT*™ CP Reagent (Guangzhou ruibo biotechnology co. LTD, Guangzhou, China). After 48 h, the cells were infected with *M. pneumoniae* (MOI = 1:100) at 37°C in the presence of 5% CO_2_ for 24 h. Cytokine production in the supernatant was then determined by ELISA.

### Statistical Analysis

Data were analyzed with SPSS 20.0. Normally distributed data were reported as the mean ± standard deviation. Classification data were compared using the Fisher’s exact and chi-square test. Numeration data were analyzed using the Student test if data distribution was normal, and measurement data were analyzed using the non-parametric test (Mann–Whitney U-test or Wilcoxon test) if data distribution was non-normal. *P* < 0.05 is statistically significant.

## Results

### Serum TNF-α and Neutrophil TLR mRNA Levels were Increased in MPP and Especially in RMPP

**Figure 1:**
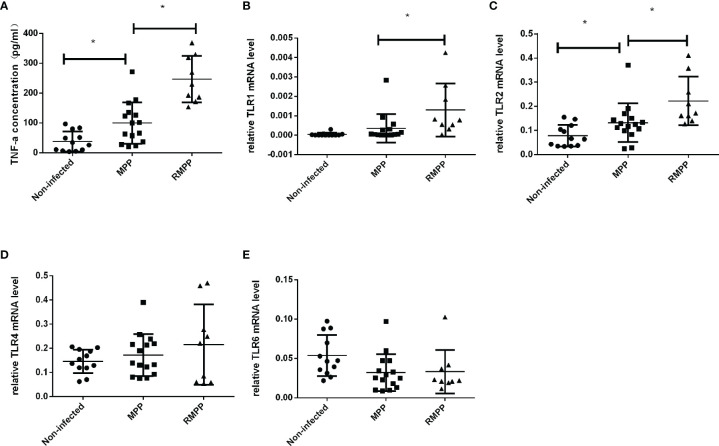
Serum TNF-α levels in the three study groups. **(A)** The levels of serum TNF-α was determined by ELISA. **(B–E)** TLR1, TLR2, TLR4, and TLR6 mRNA relative expression level in children’s blood neutrophils were determined by RT-qPCR and were normalized by human GAPDH gene. *P < 0.05 is statistically significant. MPP, *Mycoplasma pneumoniae* pneumonia; RMPP, refractory *M. pneumoniae* pneumonia.

As shown in **Figure 1A**, serum TNF-α levels were higher in children with MPP than in the non-infected group, and higher in the RMPP group than in the MPP group. The mRNA levels of TLR1, TLR2, TLR4 and TLR6 in peripheral blood neutrophils stimulated with *M. pneumoniae* were detected by RT-qPCR (**Figures 1B–E**). TLR2 mRNA expression was significantly increased in the MPP patients, and the levels were higher in the RMPP group than in the MPP group. TLR1 mRNA was also higher in the RMPP group than the MPP group. There was no significant difference in TLR4 and TLR6 mRNA expression between the three groups.

#### *M. Pneumoniae* Stimulation Enhances TNF-α Secretion and Cell-Surface Expression of TLR2 *In Vitro*

**Figure 2:**
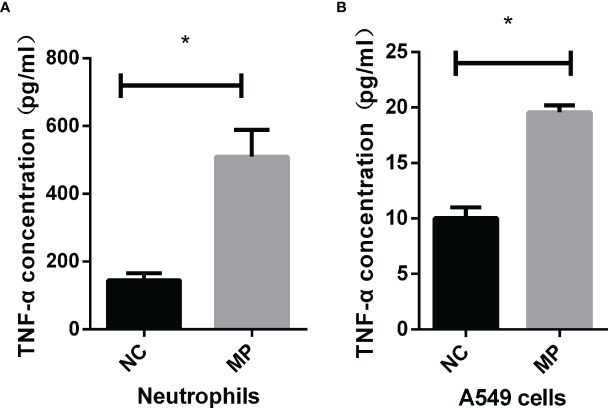
TNF-α secretion by **(A)** neutrophils and **(B)** A549 cells upon *M. pneumoniae* stimulation when compared with the NC group as assessed by ELISA. *P < 0.05 is statistically significant. NC, normal control; MP, *Mycoplasma pneumoniae*.

As shown in (**Figures 2A, B**), TNF-α production was significantly increased in neutrophils and A549 cells after stimulation with *M. pneumoniae*. To verify whether *M. pneumoniae*-stimulated cells produce TNF-α *via* TLR2, neutrophils and A549 cells were stimulated with *M. pneumoniae* and cell-surface expression of TLR2 and TLR4 was detected by flow cytometry. The results are shown in **Table 2**. TLR2 expression was significantly increased after stimulation with *M. pneumoniae* when compared with the non-infected group, whereas TLR4 showed no significant change. These above results indicated that TLR2 is upregulated after *M. pneumoniae* infection, and that TNF-α secretion may be mainly primarily enhanced by TLR2.

**Table 2:** Difference in mean fluorescence intensity between TLR2 and TLR4 in normal control group and MP stimulation group in neutrophils and A549 cells.

Cell	A649 cells		Neutrophils	
Group	TLR2	TLR4	TLR2	TLR4
Normal control group	3378 ± 760.649	902.00 ± 902.00	3945.67 ± 168.488	1117.17 ± 69.744
MP stimulation group	3760.17 ± 772.060	862.50 ± 136.488	4224.00 ± 71.125	1107.33 ± 48.306
*T*	16.337	-0.556	3.287	-0.267
*P*	<0.001**	0.602	0.022*	0.800

Data are presented as mean ± standard deviation. T, Student test; **P < 0.01, *P < 0.05, P < 0.05 is statistically significant.

MP, Mycoplasma pneumoniae.

#### TLR2 Knockdown Suppresses TNF- α Secretion

**Figure 3:**
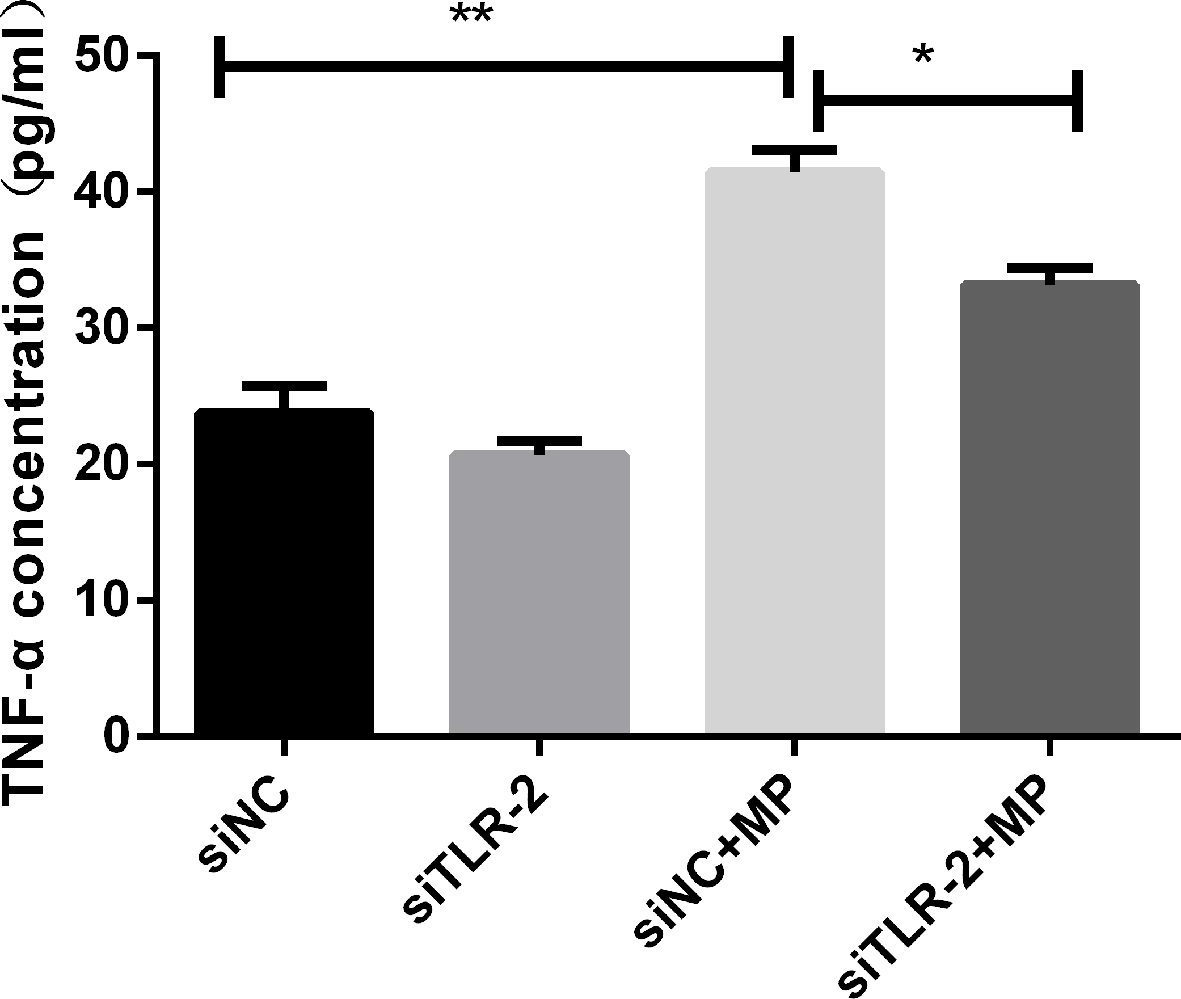
TNF-α secretion measured by ELISA in control or MP-infected A549 cells with or without knockdown of TLR2 by siRNA. **P < 0.01, *P < 0.05. *P* < 0.05 is statistically significant. siNC = A549 cells were transfected with negative control small interfering RNA (siRNA); siTLR-2 = A549 cells were transfected with siRNA targeting TLR2. MP, *Mycoplasma pneumoniae*.

After transfection of A549 cells with siRNA targeting TLR2, TLR2 mRNA expression was significantly decreased compared to that in the non-infected group. TNF-α secretion induced by *M. pneumoniae* stimulation was significantly suppressed in TLR2-silenced cells (**Figure 3**), corroborating that *M. pneumoniae*-stimulated cells secrete TNF-α through TLR2.

#### *M. Pneumoniae* Stimulation Enhances the Expression of TLR-Related Signaling Molecules

**Figure 4:**
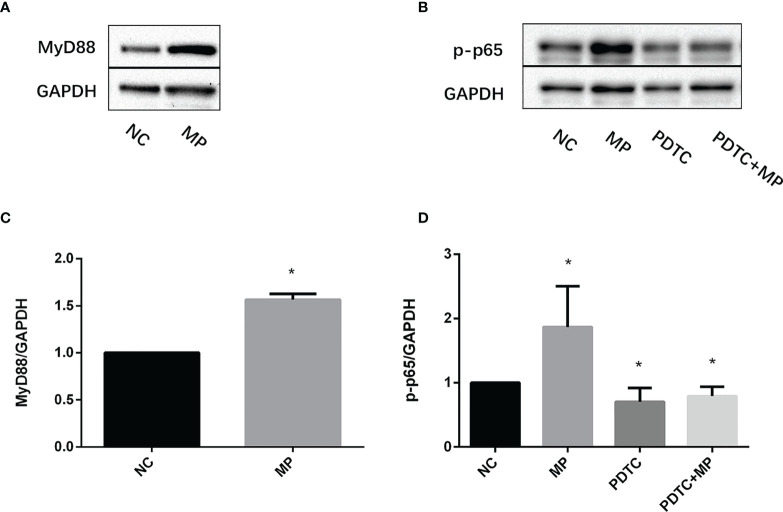
Expression changes of MyD88 and p-p65 in A549 cells stimulated with *M. pneumoniae*. **(A, B)** A representative Western blot image. **(C, D)** Densitometry analysis of band intensity was performed. Quantitative densitometry of western blots was performed using Image J analysis. The expression was normalized to GADPH, GADPH was used as a loading control. Experiments were performed in biological triplicates. *P  < 0.05 vs normal control group. *P* < 0.05 is statistically significant. NC, normal control; MP, *Mycoplasma pneumoniae*; PDTC, Pyrrolidinedithiocarbamic acid (a NF-κB inhibitor).

MyD88 and NF-κB protein(p-p65) expression was examined in A549 cells after stimulation with *M. pneumoniae* by western blotting. After *M. pneumoniae* stimulation, the protein level of MyD88 (**Figures 4A, C**), NF-κB activation status(p-p65) (**Figures 4B, D**) were increased, which confirmed the activation of MyD88 and NF-κB after *M. pneumoniae* stimulation.

#### Blockage of Myd88 or NF-κB Attenuates *M. Pneumoniae*-Stimulated TNF- α Secretion

**Figure 5:**
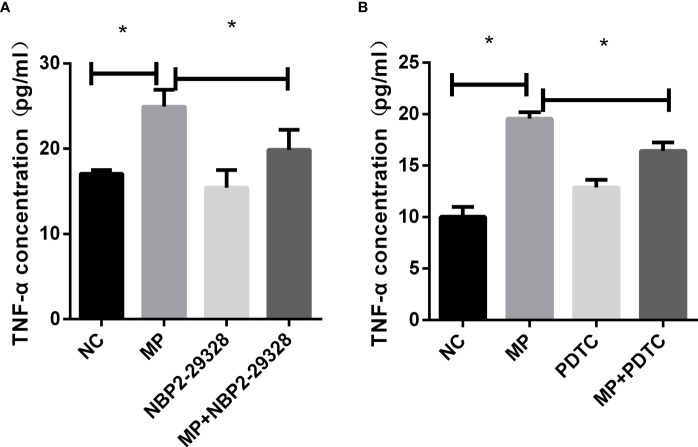
TNF-α secretion measured by ELISA in control or MP-infected A549 cells incubated with **(A)** MyD88 or **(B)** NF-κB inhibitors. *P < 0.05 is statistically significant. NC, normal control; MP, *Mycoplasma pneumoniae*; PDTC, Pyrrolidinedithiocarbamic acid, a NF-κB inhibitor; NBP2-29328, a MyD88 Inhibitor Peptide.

To confirm the role of MyD88 and NF-κB in the secretion of TNF-α, MyD88 or NF-κB were pharmacologically inhibited and A549 cells were then stimulated with *M. pneumoniae*, and TNF-α secretion was measured (**Figures 5A, B**). PDTC and NBP2-29328 significantly suppressed the increase in TNF-α secretion induced by *M. pneumoniae*, indicating that MyD88 and NF-κB play an important role in TNF-α secretion.

#### Roles of TLR2 in *M. pneumoniae*-Mediated Pulmonary Inflammation *In Vivo*

**Figure 6:**
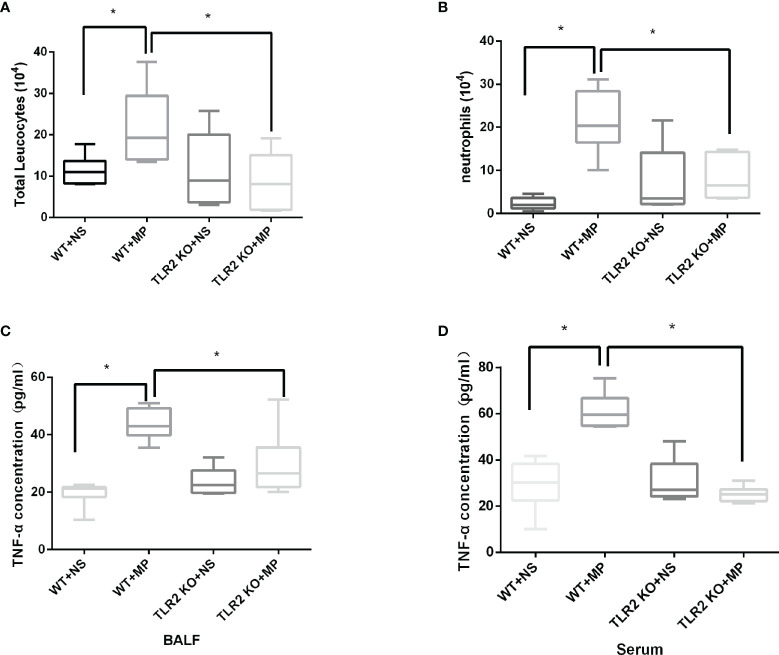
Numbers of **(A)** BALF total leukocytes, **(B)** neutrophils, and TNF-α concentration in **(C)** BALF and **(D)** serum in wild-type and TLR2 knockout mice. *P < 0.05 is statistically significant. WT, wild-type mice; TLR2 KO, TLR2 knockout mice; NS, normal saline; MP, *Mycoplasma pneumoniae*; BALF, Bronchoalveolar Fluid.

**Figure 7:**
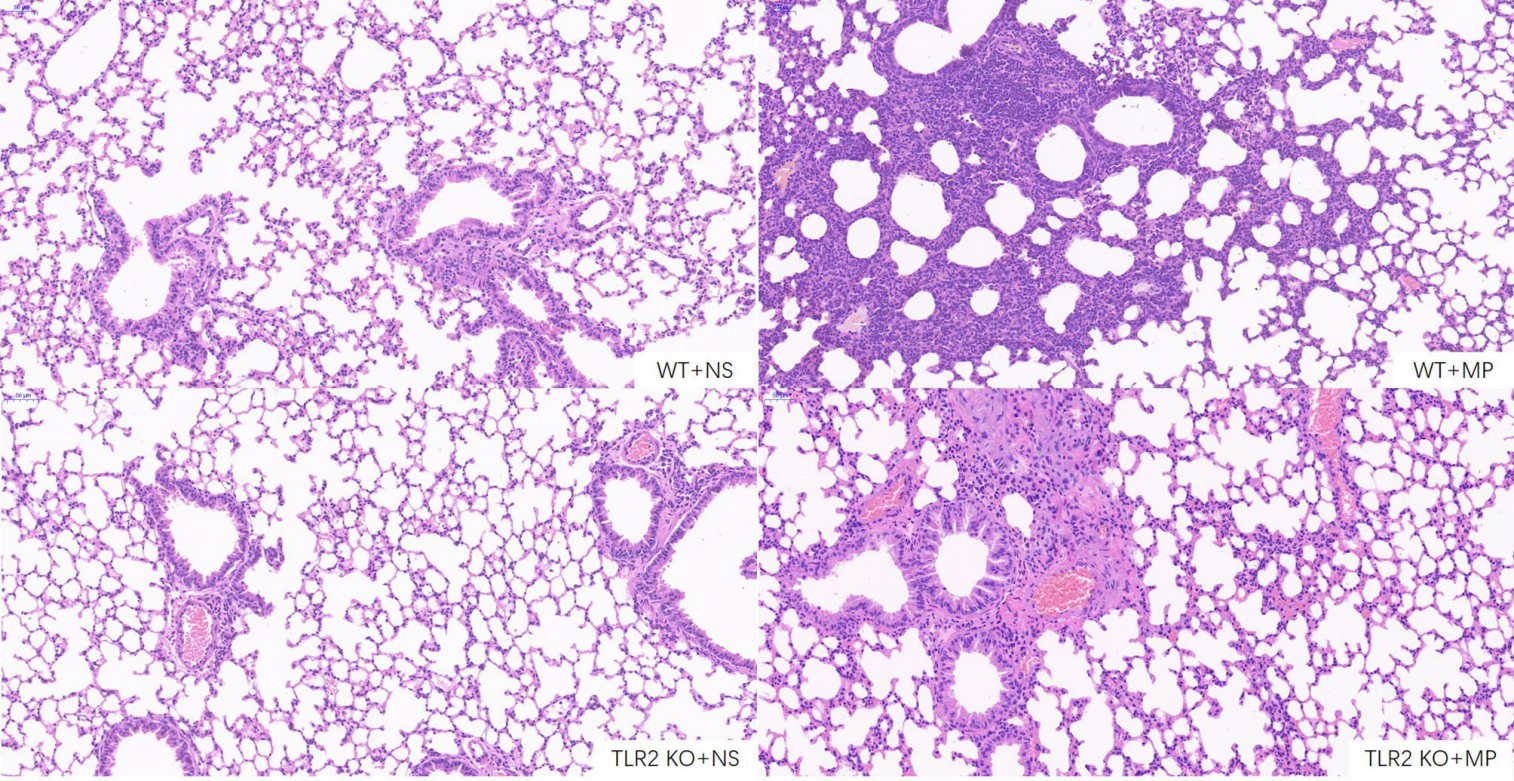
Hematoxylin and eosin staining of lung sections of wild-type and TLR2 knockout mice. Scale bar, 50μm. WT, wild-type mice; TLR2 KO, TLR2 knockout mice; NS, normal saline; MP, *Mycoplasma pneumoniae*.

Host and inflammatory responses of wild-type and TLR2-deficient mice were measured by bronchoalveolar fluid (BALF) cell count, lung histology, and cytokine release. The numbers of total leukocytes and neutrophils in the BALF were counted on day 4 after intranasal administration of *M. pneumoniae* (**Figures 6A, B**). Higher numbers of total leukocytes and neutrophils were recruited after intranasal administration of *M. pneumoniae* than normal saline in wild-type mice. Higher numbers of total leukocytes and neutrophils were recruited in wild-type mice after administration of *M. pneumoniae* than in TLR2-deficient mice, whereas there were no significant differences between intranasal administration of *M. pneumoniae* and normal saline in TLR2-deficient mice. The concentration of cytokine TNF-α in BALF and serum was measured on day 4 after intranasal administration of *M. pneumoniae* (**Figures 6C, D**). Compared to that of wild-type mice, the release of TNF-α was decreased in TLR2-deficient mice after infection with *M. pneumoniae*. The histopathological changes were assessed by hematoxylin and eosin staining of lung tissue samples collected after *M. pneumoniae* infection (**Figure 7**). After *M. pneumoniae* infection, the infected wild-type mice (WT + MP group) showed obvious histological lesions, including cell infiltration, thickening of alveolar walls, and even structural collapse, while infected TLR2-deficient mice (TLR2 KO + MP group) showed only slight histological lesions.

## Discussion

*M. pneumoniae* is a major causative agent of community-acquired pneumonia which can lead to both acute upper and lower respiratory tract inflammation, and extrapulmonary syndromes. Poor clinical outcomes can result from overexuberant inflammatory responses to infectious pathogens, which result in damage to the lungs during pneumonia (Dolinay et al., 2012). Understanding the pathogen-host interactions that result in inflammation-mediated damage to host tissues is needed to prevent and treat severe complications of pneumonia.

In some past studies, it has been found that lipoprotein is one of the main pathogenic components of *M. pneumoniae*, which can induce inflammation and pneumonia (Okusawa et al., 2004; Shimizu et al., 2005). However, most studies mainly use the purification or synthesis of the lipoprotein components of *Mycoplasma* species to confirm that TLR2 mediates this inflammatory reaction process from the *in vitro* and/or animal level. Our current study tried to investigate the influence of excessive inflammation in the development of MPP, and to evaluate the role and mechanism of TLRs in cells, animals, and populations more comprehensively.

Although adhesion to epithelial cells is the first step in the pathogenesis of *M. pneumoniae*, and epithelial cells have the function of secreting TNF-α, the occurrence of lung inflammation and systemic hyperinflammation may be mainly mediated by the inflammatory cascade of neutrophils under the co-stimulation of *M. pneumoniae* and TNF-α. We also reported in the previous period that the population of MPP, especially the RMPP, had a significant increase in neutrophils in the peripheral blood (Shi et al., 2019), as well as the increase in neutrophils in the mouse alveolar lavage fluid in the present study. It also confirmed this idea to a certain extent.

TLR2 plays a key role in the development of airway hyperresponsiveness and of chronic airway inflammation after infection. Yang et al. (2009) found that TLR2 deficiency or suppression not only prevented bleomycin-induced inflammation, but also protected from and reversed progressive pulmonary fibrosis through a reversion of the immunosuppressive microenvironment in the bleomycin-induced fibrotic tissue. Using cell experiments, Razonable et al. (2006) identified TLR-2 as a critical receptor in mediating bleomycin-stimulated pulmonary inflammation and fibrosis *via* activation of an intracellular signaling pathway that results in the translocation of NF-κB and the secretion of TNF-α and IL-1β. Eder et al. (2004) reported TLR2 as a main causative gene of asthma in European children. (Ben-Ali et al. (2004) found that abnormal mutations in TLR2 often lead to a significant increase in susceptibility to tuberculosis. At the same time, we also noticed that Shimizu et al. (2014) investigated that the changes in the secretion of inflammatory factors in macrophages derived from TLR2-/- mice after *mycoplasma* stimulation. Compared with Shimizu’s research results (Shimizu et al., 2014), we paid more attention to the role of TLR2 in MPP disease, and studied the changes in lung tissue and pathological damage of TLR2-/- mice after *M. pneumoniae* infection, and found that TLR2 knockout inhibited lung histopathological damage. This further confirms that TLR2 plays an important role in the excessive immune response caused by *M. pneumoniae*.

In addition, our study showed that levels of TLR2, MyD88, and p-p65 in A549 cells increased after *M. pneumoniae* stimulation, and TNF-α secretion induced by *M. pneumoniae* decreased significantly after inhibition of TLR2, MyD88, or NF-κB, indicating that TLR2-MyD88-NF-κB axis plays an important role in the induction of TNF-α production in MPP.

In conclusion, we cannot deny that TLR2 mediates the production of pro-inflammatory factor TNF-α and is involved in lung inflammatory injury. It may be through the TLR2-MyD88-NF-κB signaling pathway. Compared with children with non-RMPP, TLR2 expression was increased in peripheral blood of children with RMPP. the expression level of TLR2 in RMPP children’s peripheral blood was increased compared with non-RMPP children. Therefore, could the expression level of TLR2 be used as an indicator of disease severity? Maybe TLR2 related regulatory genes be specific in children with RMPP? This is an area that we need to study further.

## Data Availability Statement

The raw data supporting the conclusions of this article will be made available by the authors, without undue reservation.

## Ethics Statement

The studies involving human participants were reviewed and approved by the Ethics Committee of Nanjing Medical University. Written informed consent to participate in this study was provided by the participants’ legal guardian/next of kin. The animal study was reviewed and approved by the Ethics Committee of Nanjing Medical University.

## Author Contributions

MC, HD, DZ, and FL contributed to the conception, designed experiments, and were responsible for the whole work. YZ, HG, YFZ, YG, and SS performed experiments. YB, JX, MC, and XM analyzed experimental results and wrote the manuscript. All authors contributed toward data analysis, drafting, and critically revising the paper, gave final approval of the version to be published, and agree to be accountable for all aspects of the work.

## Funding

This research was supported by the Special funds for provincial key R & D plans of Jiangsu Province (BE2019607), the key projects of Nanjing health and Family Planning Commission(ZKX18041), the general projects of Nanjing health and Family Planning Commission(YKK18194), the funding project for the development of Nanjing medical technology (JQX15008), the project of medical talents of Jiangsu Province (QNRC2016087), the Scientific and Technological Project from the Department of Science and Technology of Nanjing (201723003), and a project of Nanjing Medical University (2017njmuzd054).

## Conflict of Interest

The authors declare that the research was conducted in the absence of any commercial or financial relationships that could be construed as a potential conflict of interest.

## Publisher’s Note

All claims expressed in this article are solely those of the authors and do not necessarily represent those of their affiliated organizations, or those of the publisher, the editors and the reviewers. Any product that may be evaluated in this article, or claim that may be made by its manufacturer, is not guaranteed or endorsed by the publisher.
