# Supplementary figures and images for: Toll-Like Receptor 2 Modulates Pulmonary Inflammation and TNF-α Release Mediated by Mycoplasma pneumoniae
Source: Front Cell Infect Microbiol. 2022 Mar 17;12:824027. doi: 10.3389/fcimb.2022.824027 (PMC8968444; doi:10.3389/fcimb.2022.824027)

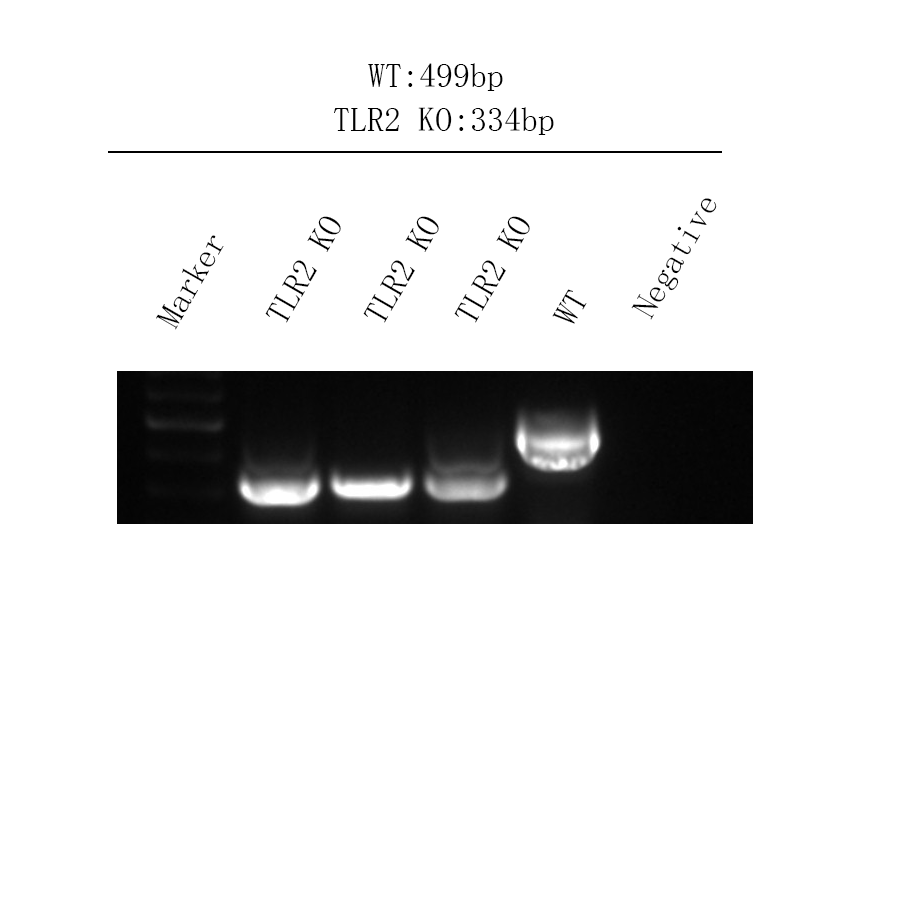

Supplement: Supplementary file 1 [file Image_1.tif]
